# Neutral and adaptive explanations for an association between caste-biased gene expression and rate of sequence evolution

**DOI:** 10.3389/fgene.2014.00297

**Published:** 2014-08-29

**Authors:** Heikki Helanterä, Tobias Uller

**Affiliations:** ^1^Department of Biosciences, Centre of Excellence in Biological Interactions, University of HelsinkiHelsinki, Finland; ^2^Department of Zoology, Edward Grey Institute, University of OxfordOxford, UK; ^3^Department of Biology, University of LundSölvegatan, Lund, Sweden

**Keywords:** polymorphism, social insects, phenotypic plasticity, neutral evolution, antagonistic pleiotropy

## Abstract

The castes of social insects provide outstanding opportunities to address the causes and consequences of evolution of discrete phenotypes, i.e., polymorphisms. Here we focus on recently described patterns of a positive association between the degree of caste-specific gene expression and the rate of sequence evolution. We outline how neutral and adaptive evolution can cause genes that are morph-biased in their expression profiles to exhibit historical signatures of faster or slower sequence evolution compared to unbiased genes. We conclude that evaluation of different hypotheses will benefit from (i) reconstruction of the phylogenetic origin of biased expression and changes in rates of sequence evolution, and (ii) replicated data on gene expression variation within versus between morphs. Although the data are limited at present, we suggest that the observed phylogenetic and intra-population variation in gene expression lends support to the hypothesis that the association between caste-biased expression and rate of sequence evolution largely is a result of neutral processes.

## Introduction

Polymorphic populations are comprised of distinct interbreeding phenotypes. These include males and females, alternative male mating morphs and different forms of resource, dispersal, or defense polymorphisms (West-Eberhard, 2003). Notably, polymorphisms also include the social insect castes, such as queens and workers. Different forms of polymorphisms vary in several respects (Box 1), but they have in common that determination and maintenance of morph-specific phenotypes involve differential expression of genes. Polymorphisms are outstanding model systems for studying the relative importance of changes in regulatory versus coding sequences (Carroll, 2005; Hoekstra and Coyne, 2007), and the interchangeability of genes and environments in phenotypic evolution (West-Eberhard, 2003; Schwander and Leimar, 2011; Uller and Helanterä, 2011).

Box 1 **Animal polymorphism**

Different forms of polymorphisms share many features, but there are also important differences that may affect how the developmental genetic regulation of their determination and function will evolve. This is an area that would benefit strongly from theory and comparative analyzes, and here we can only provide a brief summary of some morph features that may be important for the relationship between patterns of gene expression and sequence evolution.

All morphs are by definition discrete phenotypes, but the degree to which morphs differ from each other varies dramatically between systems. Queens and workers are among their more extreme polymorphisms, but even within social insects the extent to which they differ morphologically (e.g., size and shape) and physiologically (e.g., reproductive activity, lifespan) is quite variable (Bourke, 1999). In some systems where morphs represent, for example, alternative reproductive strategies, it is not necessarily the case that the average difference in gene expression between morphs is greater than differences within morphs (e.g., throughout the season). Other morphs are not even functionally different and hence might only differ consistently in gene expression during morph determination (e.g., some color or pattern morphs).

Morphs also sometimes differ genetically. The most familiar example is the sexes in mammals. It is unclear if and how the extent to which morph determination relies on the presence of specific genes versus specific environments should affect the evolution of morph-biased gene expression. Comparisons of closely related species with environment- versus genotype-dependent sex or caste determination would be informative. Related to this is the extent to which genome evolution in species with genotypic morph determination parallels that of sex chromosome evolution, which not only affects morph-specific gene content but also the extent of antagonistic selection across the genome (Connallon and Clark, 2010).

Polymorphisms can be maintained by different forms of selection. In some cases the average fitness of morphs may be equal when averaged across contexts, for example due to frequency-dependent selection. In other cases one morph is adopted under poor conditions and is therefore maintained also when it exhibits consistently lower fitness on average. The selective dynamics affect the frequency of morphs within populations and hence the strength of selection on genes with morph-specific expression or function (Van Dyken and Wade, 2010). But it is also possible that different forms of selection create particular signatures in terms of sequence evolution, for example their tendency to maintain nucleotide polymorphism within populations (Nielsen, 2005). The outcome of these processes will also be affected by the age of polymorphisms. For example, we suggest that antagonistic selection may be more common in the early stages of morph evolution when resolution of antagonism through expression patterns has not had time to evolve yet, and neutral evolution of both sequence and expression pattern more common in highly canalized morphs.

Morphs are defined at the level of the individual organism. However, in social insects where the colony, or even the supercolony, can function as an individual organism (Queller and Strassmann, 2009), it is possible that morph-biased gene expression is not analogous to, for example, sex-specific gene expression, but more similar to the differential expression of genes among tissues. Some predictions for the rate of sequence evolution of tissue-specific and morph-specific genes compared to constitutively expressed genes are shared, but others differ (in particular when not all morphs are capable of reproduction). Analogously to genes that are over-expressed in one tissue, to understand the actual strength of selection on worker-biased genes it may be necessary to understand how this expression pattern contributes to the performance of the colony (i.e., the reproductive unit).

The recent increase in availability of large scale gene expression data through microarray and whole transcriptome sequencing has facilitated quantitative and qualitative description of the developmental genetic basis of polymorphism. In social insects, caste biased expression patterns have been investigated transcriptome wide in, for example, honeybees *Apis mellifera* (Grozinger et al., 2007), bumblebees *Bombus terrestris* (Colgan et al., 2011), *Polistes* wasps (Sumner et al., 2006; Ferreira et al., 2013) and ants such as *Solenopsis invicta* (Hunt et al., 2011, 2013) and *Temnothorax longispinosus* (Feldmeyer et al., 2014). Comparisons can be made with data from other polymorphic systems, including males versus females of laboratory model species with genotypic sex determination (e.g., mice and *Drosophila* spp, (Ranz et al., 2003; Zhang et al., 2007; Mank et al., 2008; Meisel, 2011), and a variety of environmentally induced polymorphisms, such as horn polymorphism in beetles (Snell-Rood et al., 2011), feeding type polymorphism in toad tadpoles (Leichty et al., 2012), and dispersal polymorphisms in pea aphids (Purandare et al., 2014). Sequencing methods, sampling design, pooling of samples, statistical power and definitions of what qualifies as a morph biased expression pattern vary extensively among studies, and the proportions of genes or transcripts that are classified as morph-biased range from a few to several tens of percents. For example, between 7.5% (Hunt et al., 2011) and 40% (Grozinger et al., 2007) of studied genes have been classified as caste biased in social insects. In males and females, Naurin et al. (2011) describe only 1.6–2.4% of genes as sex biased in two bird species, whereas as many as 90% of genes were reported to exhibit sex biased expression in a study of *Drosophila* (Innocenti and Morrow, 2010). Approximately half of the genes in the pea aphid show a biased expression pattern according to either morph or sex (Purandare et al., 2014). However, across all these examples, very few genes, if any, are exclusively expressed in one morph. That is, gene expression patterns vary between morphs in degree, and not in an on-or-off manner.

Morph-biased gene expression has a wide range of causes and consequences that are of interest to developmental and evolutionary biologists. In the rest of this commentary we focus on the intriguing observation that worker or queen biased genes in ants and social bees appear to evolve faster at the sequence level than do genes with no expression bias (Hunt et al., 2010, 2011; Feldmeyer et al., 2014). This is not just a social insect phenomenon. For example, it has repeatedly been shown in fruit flies and mice that both male and female biased genes evolve faster than unbiased genes (for recent studies see Meisel, 2011; Assis et al., 2012; Grath and Parsch, 2012, reviewed in Parsch and Ellegren, 2013). The same pattern has also been found with respect to sex-specific reproductive functions in *Arabidopsis* (Gossmann et al., 2014), tadpole feeding morphs in spadefoot toads (Leichty et al., 2012), horn polyphenisms in beetles (Snell-Rood et al., 2011), and dispersal morphs in pea aphids (Purandare et al., 2014).

There are a number of potential explanations for these patterns. To the extent that the morphs reflect different reproductive roles, as is the case for males and females, queens and workers, and dispersing sexuals and sedentary asexuals, faster sequence evolution of biased genes can partly be explained by faster evolution of reproductive genes (Meisel, 2011; Wright and Mank, 2013). Fast evolution of reproductive genes has been attributed to sexual selection, including sexual conflict and sperm competition, which is expected to increase the rate of sequence evolution (Swanson and Vacquier, 2002). Similar arguments should apply to the various reproductive conflicts in insect societies (Rice and Holland, 1997). Rapid evolution of reproduction-related genes may also be partly due to faster evolution of tissue specific genes when the observed sex biases arise from genes that are expressed in sex-specific reproductive tissues (Meisel, 2011). Furthermore, when interpreting results of transcriptomes of whole individuals, it needs to be kept in mind that they may reflect differences in the size or composition of tissues between morphs, rather than differences in gene expression at a cell level. More generally, studies in model organisms have established that a large number of additional factors could underlie a correlation between gene expression patterns and evolutionary rates, including expression breadth, overall expression levels, DNA methylation patterns, architecture of regulatory sequences, and potential for pleiotropy (Lemos et al., 2005a; Larracuente et al., 2008; Meisel, 2011; Park et al., 2012; Warnefors and Kaessmann, 2013). Teasing these explanations apart is major challenge in emerging model organisms such as social insects. However, the correlation between morph biased expression pattern and fast sequence evolution rate remains significant even if many of these are controlled for statistically (e.g., Snell-Rood et al., 2011; Grath and Parsch, 2012; Warnefors and Kaessmann, 2013), suggesting that this relationship may be a fundamental feature of the evolution of genomes.

A recent study in fire ants suggested that caste-biased genes evolved faster at sequence level even before they became morph-biased or, indeed, before the evolution of the castes (as shown by comparisons with the solitary, monomorphic wasp *Nasonia vitripennis*; Hunt et al., 2011). Interestingly, a similar pattern was found in in toads where repeated evolution of polymorphism has taken place within a single genus (Leichty et al., 2012). A second interesting finding is that genes that vary in expression levels among morphs also seem to vary extensively in their expression levels within morphs. This is particularly well documented in fire ants (Hunt et al., 2013), and appears to occur also in polymorphic toads (Leichty et al., 2012), and sex biased genes in birds and fruit flies (Mank et al., 2007; Mank and Ellegren, 2009). As described below, both of these observations provide important pieces of evidence for addressing the role of neutral and selective explanations for associations between biased gene expression and rate of sequence evolution across the genome.

In this paper, we provide an overview of five different scenarios that predict a relationship between caste-biased gene expression and accelerated sequence evolution. We draw from both studies of sex biased gene expression in model organisms and from the diverse but less studied polymorphic organisms. We conclude that the association between caste-biased gene expression and rate of sequence evolution will be better understood if we address the contribution of selective and neutral processes for both inter-individual and phylogenetic divergence in gene expression. This requires both more detailed analyzes of individual and context-dependent variation in gene expression, and establishing whether the strength of selection on gene sequences is a cause or a consequence of changing patterns of gene expression.

## Routes to coupling of morph-biased expression and rate of sequence evolution

Contemporary patterns of variation in DNA sequence and gene expression partly reflect a mix of selective and stochastic events accumulating over evolutionary time (as well as current conditions experienced by the focal individuals). The relative importance of neutral and adaptive evolution for genome evolution is a contentious issue. DNA sequences diverge as a result of accumulation of changes that are neutral with respect to fitness or too weakly selected to be purged, but they also diverge because of repeated fixation of mutations due to selection. Similarly, divergence in gene regulation can represent both selection and drift. Here we ask how these processes can cause genes with caste-biased expression to exhibit evidence of accelerated sequence evolution.

### Neutrality

Assuming morphs are adaptive, at least some morph-bias in gene expression is a result of selection. However, in many species the number of genes that contribute to morph determination or maintenance of morph-specific phenotypes may be quite small. Thus, it is possible that a large proportion of variation in gene expression between morphs is a result of neutral evolution. Genes whose expression level is under weak selection are expected to be less precisely regulated due to accumulation of near-neutral regulatory mutations (Khaitovich et al., 2005, 2006). By chance, some of the accumulating regulatory mutations may result in morph-biased expression. Thus, morph specific expression patterns can arise through drift. This suggests that, in any given data set, morph-biased expression partly reflects a history of weak purifying selection on gene regulation. This can create a link between rate of sequence evolution and biased gene expression if genes that are under weak selection with respect to sequence are also under weak selection in terms of expression, and consequently more likely to have a drifted toward biased expression pattern than constrained genes. This is likely to often be the case given the observed correlations between gene essentiality and expression noise (Fraser et al., 2004), and expression divergence and sequence divergence among species (Lemos et al., 2005b; Zhang et al., 2007; Mcmanus et al., 2010).

**Figure 1:**
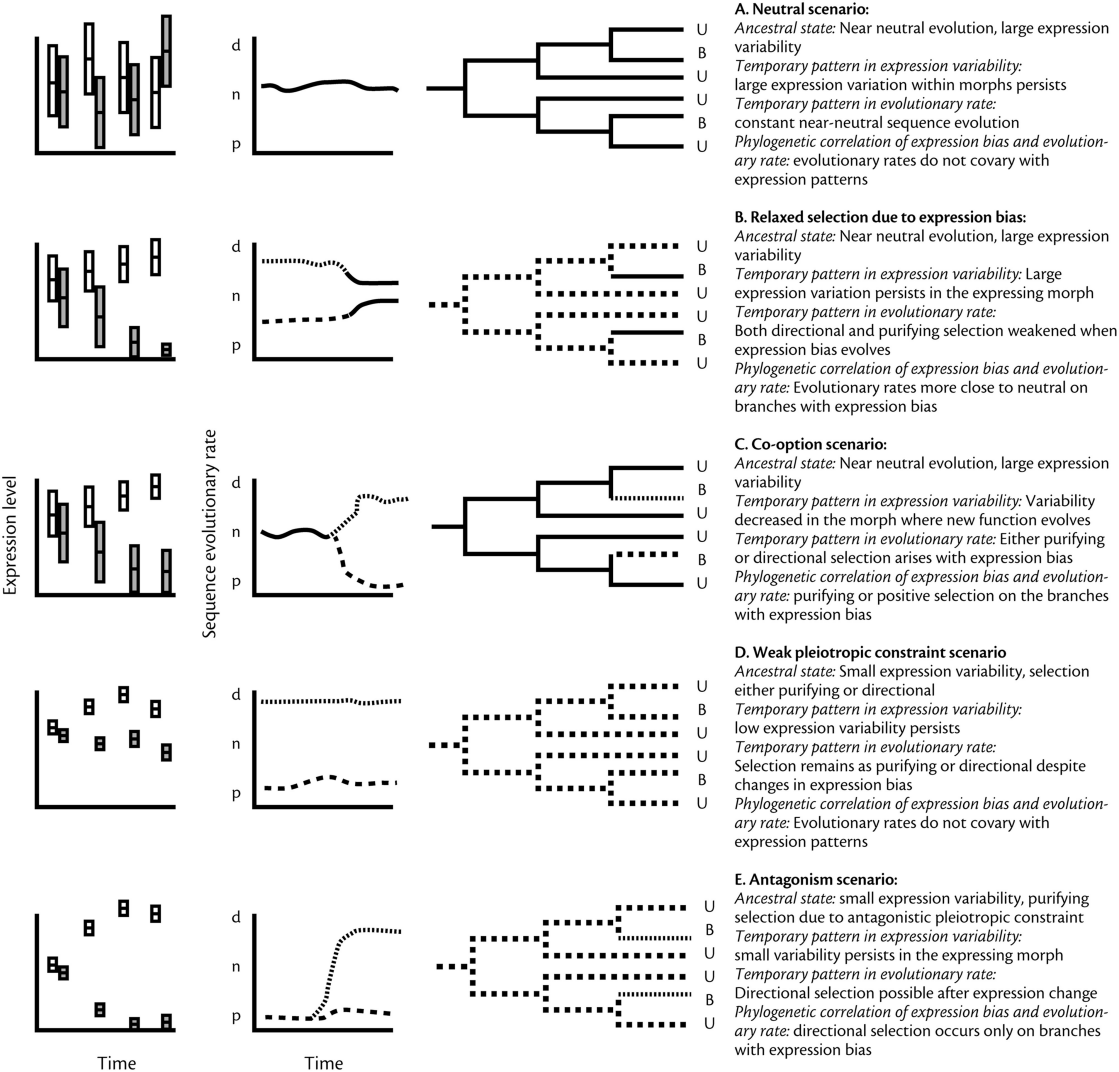
**Predicted patterns of evolutionary history of gene expression and sequence evolution for the five scenarios outlined in the text**. The left hand column shows the expected relative variation in expression within morphs and hypothetical changes in morph biased gene expression over time. White bars for morph 1 and gray bars for morph 2. The second column shows how average evolutionary rates are predicted to change over time (p, purifying; n, neutral; d, directional), and the third column shows how the patterns would be seen in a phylogeny in a group where some species show biased expression (B) for the gene in question, whereas others do not (U). Solid line, neutral rate; hatched line, purifying selection; dotted line, directional selection.

This process should result in the pattern observed in *S. invicta* (Hunt et al., 2011), where genes that presently exhibit morph-biased expression evolved faster even before the evolution of morphs or before morph biased expression pattern arose (Figure 1). Also, since it implies that regulation of expression is not under strong selection or constraint, we expect such genes to show substantial variation in their expression both between and within morphs—a pattern also shown in *S. invicta*. The temporal and phylogenetic patterns of sequence and expression evolution that would correspond to this scenario are shown in Figure 1A. Assessing neutrality of gene expression is compromised by the lack of a widely accepted neutral baseline (comparable to comparison of synonymous and non-synonymous amino acid changes in sequence data), but theory is advancing fast in this area and several alternatives have recently been proposed (Gout et al., 2010; Warnefors and Eyre-Walker, 2012; Rohlfs et al., 2014).

#### Relaxed selection due to expression bias

Interpreting a correlation between expression bias and evolutionary rate in social insects is complicated by the fact that genes expressed in workers only have indirect effects mediated through the queen genotype (Linksvayer and Wade, 2009; Hall and Goodisman, 2012). The strength of selection on worker biased genes thus depends on the genetic similarity or kinship between the worker expressing a gene, and the queen that is reproducing in the nest. Relaxed selection may also occur in polymorphic species where all morphs reproduce. A very general model of polymorphic expression suggests that, all else being equal, expression bias itself may directly contribute to evolutionary rate (Snell-Rood et al., 2010; Van Dyken and Wade, 2010). This is because once expression of a gene falls below functional levels in some individuals (e.g., one of several morphs), those genes are under direct selection in a subset of the population and hence under weaker selection than constitutively expressed genes. Thus, both directional and purifying selection become relaxed following morph-biased gene expression, which allows mildly deleterious alleles to accumulate at a higher rate than is the case for constitutively expressed genes. In one such scenario, a gene under weak selection may drift to non-detectable expression levels in one morph (assuming the strength of selection on sequence and expression are correlated as in Figure 1A), leading to further relaxation of selection and hence neutral sequence evolution (Figure 1B).

Although this is an attractive hypothesis, the extremely low number of genes that are morph-specific, rather than simply morph-biased, may suggest that few genes in fact are under relaxed selection. However, if we assume that there is a general correlation between expression level and strength of selection (Pál et al., 2001; Lemos et al., 2005a; Meisel, 2011), or that genes with a low expression level may be below a threshold value for being functional, the logic holds for genes with non-zero expression. Consistent with these predictions, genes expressed in rarer morphs of pea aphids appear to evolve faster due to relaxed purifying selection than genes biased toward the more common morphs (Purandare et al., 2014). Furthermore, the findings that both queen biased and worker biased genes evolve faster than unbiased genes (Hunt et al., 2010, 2011) suggests that fast evolution is not only due to reproductive genes (expressed in queens) evolving fast, which is broadly consistent with the general theory of relaxed selection. Nevertheless, widespread positive selection on worker biased genes in honeybees (Harpur et al., 2014) suggests that relaxed selection is not necessarily a major force limiting adaptive evolution of genes with worker biased expression.

This relaxation of selection due to gene copies in non-expressing or non-reproducing individuals being invisible to selection should apply to all genes with extreme expression bias and not only those that drift to this situation. For genes that are under positive selection before morph bias evolves, evolution could slow down and approach neutrality due to morph bias, whereas genes historically under purifying selection could start accumulating mutations and shift toward neutrality due to weakened purifying selection with morph bias. As a result, many different patterns of historical signatures of sequence evolution are possible.

### Selection

Consistent stabilizing (purifying) or directional (positive) selection can generate both slower and faster rates of sequence evolution compared to the neutral expectation. Harmful mutations in genes whose sequence is essential for organismal function are rapidly purged, resulting in slow rates of evolutionary change. On the other hand, mutations in genes that cause functional changes to phenotypes can be consistently and repeatedly selected if conditions change, such as the case in evolutionary “arms races.” Thus, if these patterns of selection covary with expression patterns, it could contribute to the observed relationship between caste-biased gene expression and the rate of gene sequence divergence.

#### Co-option of neutral genes to morph specific function

Genes with high rate of sequence evolution due to weak purifying selection may not only drift toward morph-biased expression, as described above, but also be more likely to become co-opted for morph specific functions. This is because weak selection enables the accumulation of genetic variation that can become functional in novel contexts (True and Carroll, 2002). Co-option can potentially occur during morph evolution. Alternatively, genes may become morph biased after the evolution of morphs even if they did not play a role in their original divergence. This has potential implications for the evolution of both the sequence and regulation of those genes. Both positive and purifying selection following co-option are possible and can make the rate of sequence evolution change from near-neutral toward faster or slower or, comparing site by site, increase both the proportions of sites under positive and purifying selection, respectively (Figure 1C).

In this scenario, following evolutionary rates of gene sequences over time should reveal a change from expectations of neutrality toward signatures of selection. Consequently co-opted genes should contribute to the observed correlation of expression bias and fast sequence evolution only through those genes that became positively selected following co-option, as only these genes continue to evolve fast. At the level of expression, co-option of historically “near-neutral” gene sequences should result in further selection for precise gene regulation and hence a reduction in expression noise over evolutionary time in lineages with morphs (Figure 1C). The role of co-option in the evolution of morph biased gene expression has not been directly studied in social insects, and doing so requires more information on the extent to which morph-biased genes also have morph-biased fitness effects. For example, studies showing that weak selection on sequence precedes morph biased expression (Hunt et al., 2011; Leichty et al., 2012) have not demonstrated that the subsequent expression bias reflects morph specific function rather than continued weak selection. In contrast, positive selection of worker biased genes in honeybees is also consistent with a co-option scenario, but the historical data on evolutionary rates before caste biased expression evolved is lacking (Harpur et al., 2014). Outside social insects it has been observed that up-regulated expression in one morph is linked to higher fitness effects in that morph (Connallon and Clark, 2011; Hall and Goodisman, 2012), but it is unknown whether the fitness effects caused selection for biased expression, or if biased expression arose first followed by compensatory changes to maintain morph fitness.

#### Evolution under weak pleiotropic constraint

Genes typically have multiple functional targets, which may constrain their evolution. Functional constraint contributes to the overall strength of selection on sequence and expression and is therefore implicit in much of what has already been discussed. However, the literature also emphasizes a more constructive role of weak pleiotropy where it directly causes particular fast evolving genes to become morph biased. Genes that are expressed in a context or tissue specific manner (Duret and Mouchiroud, 2000; Zhang and Li, 2004), are likely to have low number of interactions with other gene products (e.g., Assis et al., 2012) and therefore be free to evolve under directional selection. Furthermore, it has been suggested that genes that have a regulatory architecture that allows precise regulation, which also decreases pleiotropic constraint, are more likely to exhibit context sensitive expression patterns (Grishkevich and Yanai, 2013), such as morph biased expression. While the correlation of pleiotropic constraint and expression pattern has not yet been tested in social insects, it is supported by sex-specific gene expression patterns in mice, chicken and fruit flies, where sex biased genes appear to exhibit weak pleiotropic constraints (Mank et al., 2007; Meisel, 2011; see also below). If precisely regulated genes, with potentially low pleiotropic constraint, are more likely to evolve morph biased expression patterns, this should create a consistent positive correlation between morph biased expression and high rate of sequence evolution, caused by positive selection, over evolutionary time (Figure 1D).

Fast sequence evolution due to positive selection has been shown to occur in worker biased genes in the honeybee (Harpur et al., 2014), in caste biased genes in seven ant genomes (Roux et al., 2014) and male biased genes in *Drosophila* (reviewed in Wright and Mank, 2013). However, additional data is necessary before this can be taken as support for rapid evolution due to relaxation from pleiotropic constraints. If fast evolving genes with expression bias are indeed only weakly constrained by pleiotropy, we expect their evolutionary rate to be high already before the biased expression pattern evolved. Furthermore, under highly precise regulation we expect relatively low expression variation among individuals within morphs. Consistent directional selection is thought to be rare, but may be particularly likely for genes involved in reproduction, immunity and social and reproductive conflicts (Swanson and Vacquier, 2002; Summers and Crespi, 2005; Obbard et al., 2009). Because many morphs, notably sex and caste, have different reproductive functions the morph biased genes that evolve fast under directional selection may largely be composed of reproductive and conflict related genes. This could also explain why such genes evolve fast before they become morph biased (or before morphs evolve). In social insects such genes may be found among genes involved in recognition and responses to hormones, as suggested by Roux et al. (2014). Outside social insects, genes with a conserved male biased expression, likely to be involved in reproductive function and often expressed in sex specific tissues, have been shown to evolve faster than other sex biased genes in *Drosophila* (Grath and Parsch, 2012). The often narrow tissue wide expression profiles of sex biased genes may also support that genes without pleiotropic effects are more likely to become morph biased, but without temporal data it is difficult to tease apart what is cause and consequence for this association.

#### Morph antagonistic selection

One source of antagonistic selection is when an allele has beneficial effects on one morph but negative effects in another morph. This form of antagonistic selection has been discussed frequently with respect to sex biased gene expression (Rice and Chippindale, 2001; Morrow et al., 2008; Innocenti and Morrow, 2010), but here we emphasize that the same logic applies to any polymorphism, including social insect castes (see Hall et al., 2013; Holman, 2014, for specific models on caste antagonistic selection). If an allele has opposite fitness effects in two or more morphs, selection should favor suppression of expression in the morph(s) where it has negative consequences (Rice and Chippindale, 2001). Following the evolution of suppression of gene expression, antagonistic pleiotropy is relaxed, which enables genes to respond to directional selection and exhibit fast sequence evolution (Gadagkar, 1997). This follows the general logic described above, but under this scenario the changes in expression pattern and evolutionary rates are predicted to occur concurrently, i.e., sequence evolution accelerates when biased expression evolves (Figure 1E). Also, genes under positive selection should be highly regulated (Fraser et al., 2004; Wang and Zhang, 2011), and thus vary relatively little in their expression pattern within each morph. Many sex biased genes do evolve under positive selection (reviewed in Wright and Mank, 2013), and positive selection in worker biased genes has recently been demonstrated in the honeybee (Harpur et al., 2014). The expression history of these genes, and the timing of possible changes in evolutionary rates, is largely uncharted. Support for the theory would require data showing that these genes began to evolve under positive selection following the evolution of morph-biased expression.

Actual mapping of loci with antagonistic fitness effects is currently out of reach for any social insect system, but studies have recently been conducted in *Drosophila* (Innocenti and Morrow, 2010; Parsch and Ellegren, 2013). Only a minor proportion of genes with sex biased expression showed sexually antagonistic fitness effects in a hemiclonal analysis (Innocenti and Morrow, 2010), which suggests that ongoing selection for suppression is not a major explanation for biased gene expression. However, there are several reasons why some of the antagonistic fitness effects may go undetected in such coarse scale analyses (outlined in Parsch and Ellegren, 2013), and the contemporary lack of antagonistic fitness variation in sex biased genes could be a signal of a resolved ancestral conflict (Innocenti and Morrow, 2010). Because a considerable proportion of unbiased genes appear to have antagonistic fitness effects (Innocenti and Morrow, 2010), it is also possible that constraints such as intersexual genetic correlation may limit an evolutionary response to sexual antagonism in terms of biased expression. Alternatively, alleles at unbiased loci that show antagonistic fitness effects may have arisen so recently that the resulting conflict has not yet been resolved through morph-biased suppression of expression.

#### Summary of scenarios

It is important to keep in mind that even when the scenarios make mutually exclusive predictions (summarized in Figure 1), they still represent processes that can co-occur and overlap. For example, if antagonistic fitness drives the system to morph-specific expression, this leads to relaxation of selection as well. Similarly, co-option and relaxed selection can be seen as alternative interpretations of a similar process that on the one hand allows exploration of the phenotypic space and on the other hand may lead to accumulation of slightly harmful mutations and “polymorphism load.” Finally, although weak pleiotropic constraint can be viewed as the principal reason for why some genes become morph biased in their expression, it is also an important determinant of evolutionary rates in other scenarios simply by dictating the overall selection on both gene sequence and expression.

## Discussion

Recent research provides partial support for several of the scenarios described above, but the data on social insects are still very limited and it is unknown how much can be generalized from other polymorphic systems (Box 1). It is likely that several processes contribute to some extent. Thus, the empirical task is assessing the relative contribution of the different processes rather than forcing a single explanation to any given pattern. We suggest that doing so relies on two critical types of data—gene expression variation between and within species—both of which are limited in published studies to date.

First, phylogenetic mapping of the rates of sequence evolution and patterns of gene expression (Figure 1) is necessary for revealing the temporal order of changes in gene expression and sequence divergence. To date, comparisons are typically weak in terms of phylogenetic rigor (e.g., making use of two-species comparisons), and availability of a number of relevant contrasts is clearly a major challenge for future work. With more species, mapping rates of sequence divergence on a phylogenetic tree can determine whether fast-evolving genes are more likely to show morph-biased expression than slow-evolving genes. In cases where ancestral monomorphic populations are extant, such comparisons can be carried out by comparing evolutionary rates in lineages with and without morphs (e.g., Leichty et al., 2012), although in many cases replication is limited by the number of independent origins of morphs. For the advanced eusocial Hymenoptera, the number of independent origins of morphs is a serious limiting factor—but social taxa that comprise multiple origins of sociality, such as halictid bees or snapping shrimps, could be fruitful model systems for replicated studies. For cases where number of independent replicates is limiting, or where monomorphic outgroups are unavailable, the phylogenetic reconstruction of expression patterns has to be carried out gene-by-gene (see e.g., Grath and Parsch, 2012). Making the comparisons at a relevant phylogenetic scale can reveal both the effects of idiosyncratic features of specific polymorphic taxa, and possible convergent features shared across independent evolutionary origins of polymorphisms.

Furthermore, separating weak purifying selection from positive selection as causes of fast sequence change places demands on sequence data. Simple summary statistics such as the average *dn/ds* ratios per gene can reveal interesting patterns of average evolutionary rates but are unlikely to capture the complexity of the process. McDonald-Kreitman type tests are a more powerful and suitable method for detecting genes under positive selection (see e.g., Harpur et al., 2014 for a recent example) when a small number of taxa are analyzed. Furthermore, given that some of the scenarios predict concurrent changes in both the strength of positive and purifying selection, investigating site specific signatures of selection e.g., using maximum likelihood methods (Yang, 2007) in larger phylogenetic data sets might be necessary for thoroughly teasing apart the contributions of all the different processes (Nielsen, 2005).

Second, we suggest that it will be necessary to establish the patterns of variation among individuals within morphs for teasing apart adaptive and non-adaptive scenarios. This is because the processes that drive the rate of sequence divergence phylogenetically are also expected to generate different patterns of variation in gene expression among individuals within populations and species. Large expression variation among individuals in morph-biased genes would support the idea that genes become morph-biased because they are under relatively weak selection. In contrast, if genes evolve fast as a result of directional selection, this should be associated with precise gene regulation and hence biased genes should exhibit low expression variation within and between individuals of a given morph. Individual-level data on gene expression therefore provide one potential source of information that can help to evaluate the reasons for biased expression, which also sets demands for replication and careful study design for future studies. Given the large size of many social insects, replication at an individual (see e.g., Morandin et al., 2014) and tissue level (Johnson et al., 2013) should be feasible.

**Figure 2:**
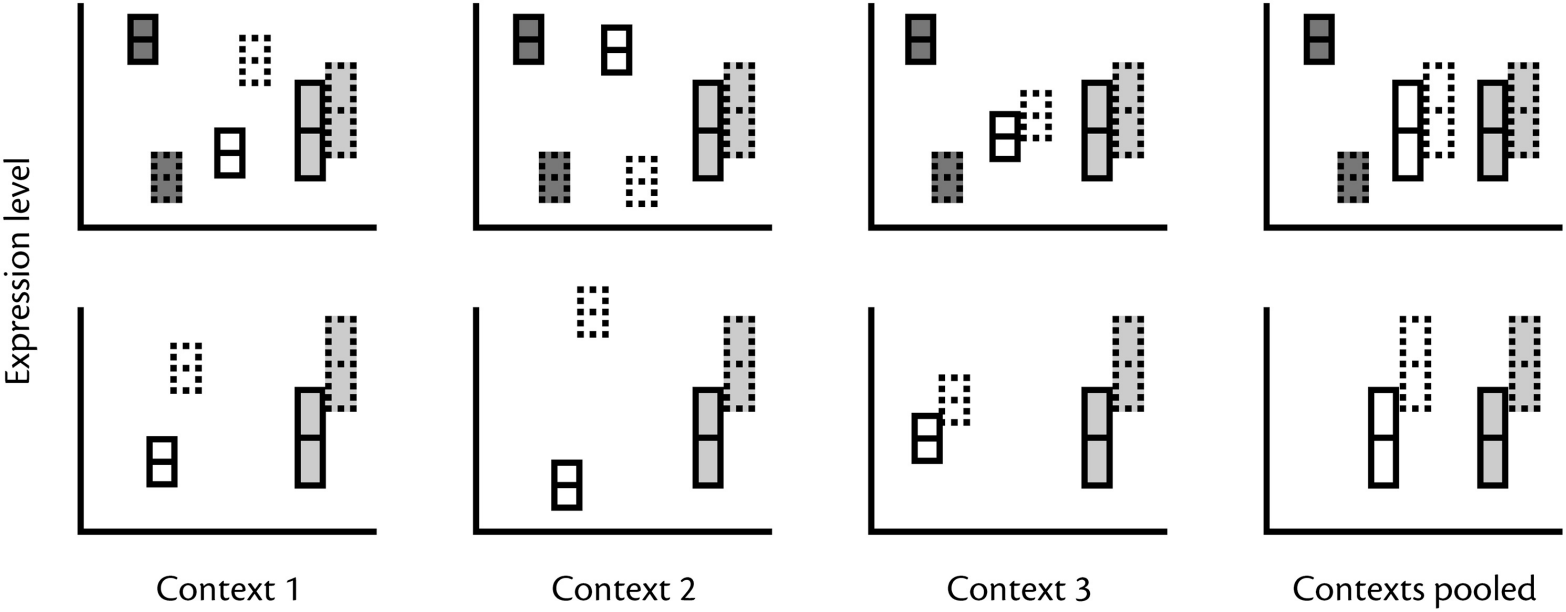
**Why variability in expression pattern may be difficult to interpret**. Boxes with solid and dotted outlines refer to expression in two different morphs. Top row: Within contexts gene expression that are consistently morph biased (dark gray boxes) or morph biased in a context-dependent manner (e.g., body size, age, past or current social experience) (white boxes) will show a morph biased pattern. In contrast, pooling samples across contexts makes context-dependent expression indistinguishable from expression variability *per se* (light gray boxes). Bottom row: In pooled samples genes regulated according to context (white boxes) are indistinguishable from genes with an expression pattern that is un-biased but highly variable (gray boxes).

Unfortunately, interpretation of gene expression variation is difficult. On the one hand, variation may represent lack of precise regulation, which causes noisy expression (Fraser et al., 2004). On the other hand, gene expression data may show substantial variation simply because of variable external or internal states not controlled during data collection (Figure 2). While it has been shown that expression is inherently noisier in non-essential genes in model organisms such as yeast, interpreting patterns of expression variability that underlie complex phenotypes is far from straightforward given the large numbers of genes that exhibit context-dependence that is unrelated to morph-specific function. For example, the proportions of genes that are differently expressed across life stages (Ometto et al., 2011; Perry et al., 2014), social environments (Manfredini et al., 2013), and genotypes (Nipitwattanaphon et al., 2013) are sometimes comparable in magnitude to morph-biased proportions. It has also been shown in studies focusing on single genes, such as vitellogenin, that caste bias is sensitive to seasonal and contextual variation (Azevedo et al., 2011, Libbrecht et al., 2013; Morandin et al., 2014). Furthermore, factors such as individual condition in *Drosophila* (Wyman et al., 2010), behavior in zebrafish (Rey et al., 2013), presence of social and sexual stimuli in swordtails (Cummings et al., 2008), and abiotic environmental conditions (Yampolsky et al., 2012) have been demonstrated to co-vary with expression patterns. These results suggest that without proper replication it cannot be assumed that all observed variation within morphs is stochastic and a sign of weak regulation. Also the observation that morph bias varies extensively between life stages (Ometto et al., 2011) and tissues (Mank et al., 2008) suggests that the more we understand the causes of variation in expression patterns, the fewer genes will be consistently classified as morph biased (Meisel, 2011).

Importantly, assessing any adaptive scenario for gene expression variation is only possible when compared against a suitable neutral expectation. While the neutral evolution of morph biased expression patterns has been directly assessed in only a few cases, studies of selection acting on gene expression patterns in general may shed some light on this issue. There are several recently suggested neutral scenarios in the literature (Gout et al., 2010; Warnefors and Eyre-Walker, 2012; Smith et al., 2013; Rohlfs et al., 2014) but empirical studies that address neutral expectations have focused on species divergence in gene expression and not morph-biased expression. It has been suggested that the factors that cause gene expression to diverge among species (e.g., non-essentiality) also expose genes to evolve context specific expression patterns (Grishkevich and Yanai, 2013). Whether general conclusions about selection on gene expression also apply to caste specific patterns remains an open question, but we suggest that they may very well do. This is supported by the finding of enriched signatures of adaptive regulatory evolution in genes underlying worker behavioral plasticity in honeybees (Harpur et al., 2014), and the extensive diversification of regulatory elements in social insects in general (Simola et al., 2013). Overall, the evidence for selection on gene expression is mixed, but a prevalence of stabilizing selection has been suggested (Gilad et al., 2006; Khaitovich et al., 2006; Warnefors and Eyre-Walker, 2012). In contrast, the relatively large turnover in the set of morph biased genes [caste biased genes between two species of *Polistes* paper wasps (Ferreira et al., 2013), between two species of *Cryptotermes* termites (Weil et al., 2009), sex biased genes among species of *Drosophila* (Metta et al., 2006; Zhang et al., 2007; Jiang and Machado, 2009; Assis et al., 2012) and between zebra finch *Taeniopygia guttata* and common whitethroat *Sylvia communalis* (Naurin et al., 2011)] supports that neutral processes play a large role, implying that genes acquire or lose morph biased expression largely due to drift. This is consistent with studies comparing a small numbers of genes in closely related species that have shown that caste biases may be evolutionarily labile (Weil et al., 2009; Morandin et al., 2014).

## Conclusions

Recent data suggests a relationship between the rate of sequence evolution and morph-biased gene expression in social insects and other polymorphic taxa, but its causes remain poorly understood. Morph-biased genes can evolve faster for several reasons. We suggest that the majority of morph-biased genes are under relatively weak selection, which can also explain why those genes evolve faster before the evolution of morphs. This suggests that adaptive scenarios should be treated with caution unless further supporting evidence can be provided. However, we also suggest that genes that ancestrally have been under weak selection, and therefore show high accumulation of mutations, may be co-opted in morph evolution and hence continue to evolve fast because of directional selection. Alternatively, co-option can lead to a reduction in the rate of evolution because of purifying selection following the onset of morph-biased expression. There are therefore several different possible genomic signatures of the evolution of morphs. Distinguishing between adaptive and (near-)neutral scenarios for the coupling of the rate of sequence evolution and morph-biased expression will require data to be replicated in several dimensions (individuals, contexts, morphs, species) at a level that is only now beginning to be possible in any taxa, including social insects. Many of the reported correlations to date are weak, and the patterns are likely to be refined by carefully assessing different functional classes of genes, more detailed studies of tissue specific expression, and studies that directly assess the evolution of gene regulation.

### Conflict of interest statement

The authors declare that the research was conducted in the absence of any commercial or financial relationships that could be construed as a potential conflict of interest.
